# An experimental and computational investigation of the cyclopentene-containing peptide-derived compounds: focus on pseudo-cyclic motifs via intramolecular interactions

**DOI:** 10.1098/rsos.240962

**Published:** 2024-10-09

**Authors:** Joanna Bojarska, Martin Breza, Paweł Borowiecki, Izabela D. Madura, Krzysztof Kaczmarek, Zyta M. Ziora, Wojciech M. Wolf

**Affiliations:** ^1^ Chemistry Department, Institute of Ecological and Inorganic Chemistry, Technical University of Lodz, 116 Zeromskiego St., Lodz 90-924, Poland; ^2^ Department of Physical Chemistry, Slovak Technical University, Radlinskeho 9, Bratislava SK-81237, Slovakia; ^3^ Laboratory of Biocatalysis and Biotransformation, Department of Drugs Technology and Biotechnology, Faculty of Chemistry, Warsaw University of Technology, 75 Koszykowa St., Warsaw 00-662, Poland; ^4^ Faculty of Chemistry, Warsaw University of Technology, 3 Noakowskiego St., Warsaw 00-664, Poland; ^5^ Institute of Organic Chemistry, Faculty of Chemistry, Lodz University of Technology, 116 Zeromskiego St., Lodz 90-924, Poland; ^6^ Institute for Molecular Bioscience, The University of Queensland, St Lucia QLD 4072, Australia

**Keywords:** modified amino acids, cyclopentene, single crystal, Hirshfeld surface, energy frameworks, DFT

## Abstract

Conformational flexibility is one of the main disadvantages of peptide-based compounds. We focus on their molecular ‘chameleonicity’ related to forming pseudo-cyclic motifs via modulation of weak intramolecular interactions. It is an appealing strategy for controlling equilibrium between the polar open and the nonpolar closed conformations. Within this context, we report here the crystal structure of the (*R*)-(2-*tert*-butoxycarbonyl)amino-1-oxo-3-phenyl)propyl)-1-cyclopentene (**1**), synthesis of which in high yield was achieved by a facile multi-step protocol. Our Cambridge Structural Database (CSD) overview for the peptide-based crystals revealed the exclusivity of this compound from the viewpoint of the unusual pseudo-bicyclic system via C–H^…^O and C–O^…^π interactions, in which cyclopentene shields the amide bond. Notably, cyclopentene as a bioisostere of proline is an appealing scaffold in medicinal chemistry. An extensive combined experimental and computational study provided more profound insight into the supramolecular landscape of **1** with respect to similar derivatives deposited in the CSD, including the tendency of cyclopentene for the generation of pseudo-cyclic motifs through weak H-bonding and π-based intramolecular interactions. These weak interactions have been examined by either the quantum theory of ‘atoms-in-molecules’ (QTAIM) or complex Hirshfeld surface methodology, including enrichment ratios, molecular electrostatic potential surfaces and energy frameworks. In all analysed crystals, all types of H-bonded motifs involving cyclopentene are formed at all levels of supramolecular architecture. A library of cyclopentene-based H-bonding synthons is provided. A molecular docking study depicted vital interactions of cyclopentene with key amino acid residues inside the active sites of two prominent protein kinases, uncovering the therapeutic potential of **1** against breast cancer. To a large extent, dispersion forces have significance in stabilizing the supramolecular structure of both ligand and bio-complex ligand–protein. Finally, the satisfactory *in silico* bio-pharmacokinetic profile of **1** related to drug-likeness and blood–brain barrier permeation was also revealed.

## 1. Introduction

The rational design of crystal structures with controllable properties that have practical and scientific relevance is a crucial target in the development of next-generation drugs.

In recent years, a significant increase in peptide-derived drugs has been seen, and they now represent *ca* 10% of the total drug market owing to their unique nature [1–3]. Peptides are very versatile molecules with great structural and functional diversity. They combine features of small chemical molecules and biological compounds; hence, their role is difficult to imitate by traditional compounds. Nevertheless, peptides suffer drawbacks such as low stability or high conformational freedom. On the other hand, these limitations can be overcome by rapid biotechnological progress and structural modifications [1,4–6]. Cyclopentene, as a bioisostere of proline, is a perfect mimetic of a natural moiety. In this context, the carbon–carbon double bond has several advantages, such as similar geometry to a peptide bond but in a planar-locked configuration, greater stability, and less susceptibility to degradation by proteases [7]. The cyclopentene ring has relevance in ligand–receptor interactions [8]. It is also a universal building block in the synthesis of bioactive compounds. Cyclopentene is a popular and important scaffold in natural products and biactive molecules, *inter alia* abacavir, adecypenol, neoplanocin A, vibralactone A, laurokamurene B, cryptophomic acid, cryptodiol and cryptotriol [9–13]. Therefore, cyclopentene-containing peptide-based compounds are in line with the demand of modern medical chemistry and crystal engineering for the intelligent design of more effective drugs, cosmeceuticals and biomaterials. The aim of peptidomimetics design is the stabilization of their bioactive conformation. The balance between rigidity and conformational flexibility is an important aspect of drug design. Molecules that are too rigid may exhibit better *in vivo* activity but worse pharmacokinetic parameters.

Crystal engineering employing weak noncovalent interactions has gained significant interest in bio-pharmacy [14]. Smart design can efficiently be realized through weak noncovalent interactions, which play a pivotal role in biological systems [15]. The interactions amide–π, apart from C–H^…^O and π^…^π, are commonly found in bio-complexes [16] and can play a vital role in bonding interactions [17]. Lone-pair^…^π (O^…^π) interactions have a pivotal function in stabilizing DNA and protein structures and participating in the formation of the DNA–protein complexes [18]. On the other hand, C–H^…^O interactions play a vital role in understanding molecular conformation [19] and conformational equilibrium [20]. Modulation of intramolecular interactions forming pseudo-cyclic systems is an attractive new approach to control conformation or interactions with the appropriate receptor precisely [21,22]. Pseudo-cyclic systems via intramolecular interactions may be valuable scaffolds in drug discovery by mimicking the bonding and electron delocalization present in similar natural entities. The incorporation of an intramolecular H-bonding motif has benefits in the design of drugs with improved properties. It can alter solubility, permeability, pharmacokinetic and pharmacodynamic properties, as well as protein binding affinity [22–24]. Moreover, owing to its planarity, such a motif can be employed in the pseudo-ring replacement strategy (with incorporated various rings) to modulate conformation. Intramolecular hydrogen bonds forming pseudo-cyclic motifs increase both *in vivo* stability and permeability across cell membranes and into molecular targets in cells, and help the molecules dynamically adapt to the environment, i.e. behave like ‘molecular chameleons’. In this sense, intramolecular hydrogen bonds provide an environment-dependent shielding of the polar group (in this case a peptide bond) when the closed/cyclic form is preferred in the apolar environment (inside a cell membrane) and the open one in an aqueous medium [25]. Studies have proved that molecules that dynamically shield or expose polarity may have higher cell permeability or aqueous solubility respectively, unlike their rigid analogues [26,27]. Nevertheless, strategies for designing molecular chameleons are still not well understood. Moreover, although molecular chameleons are related to macrocycles and molecules beyond the so-called ‘Lipinski’s rule of 5’, some strategies, including those described in this paper, could be used in designing peptide-based molecules with chameleonic properties.

**Scheme 1.**
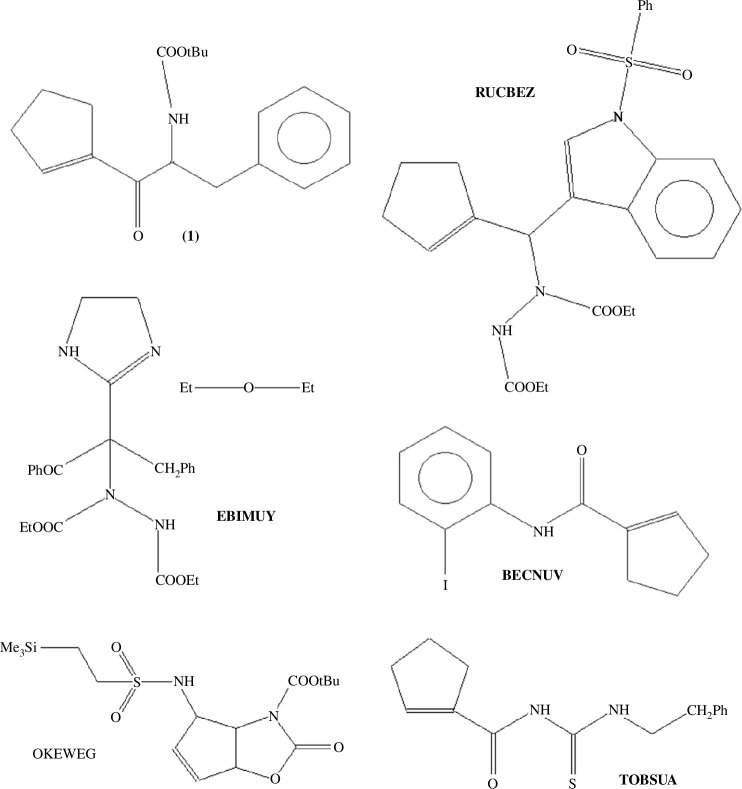
Molecular structures of compounds studied in this paper.

In light of this background and in continuation of our ongoing research on the supramolecular exploration of peptide-based compounds concerning pharmacologically relevant structural modifications [28–35], we report herein the synthesis and crystal structure of a newly modified phenylalanine-containing cyclopentene, namely (*R*)-(2-*tert*-butoxycarbonyl)amino-1-oxo-3-phenyl)propyl)-1-cyclopentene), here called **1**, to investigate shielding of the peptide bond by weak intramolecular interactions and their influence on chameleonic behaviour. According to X-ray diffraction data, compound **1** can adopt an intriguing pseudo-bicyclic structure. Notably, such systems are not common, as can be seen in the Cambridge Structural Database (CSD) [36]. Motivated by these findings, we chose a set of similar peptide-based molecules, illustrated in scheme 1 (and the electronic supplementary material, figure S1; also see §2.4 for more details), as good models for the exploration of the influence of weak noncovalent interactions on the supramolecular topology and properties. A computational analysis of the intermolecular interactions provides a rationalization based on density functional theory (DFT) calculations, the quantum theory of atoms-in-molecules (QTAIM), full interaction maps, Hirshfeld surface (HS) analysis with derived enrichment ratios, molecular electrostatic potential (EP) surfaces, and the energy frameworks (EF). Furthermore, our project fits in with the ‘pharmacophore-merging strat)egy’. The combination of the amide group, phenylalanine, and cyclopentene scaffold offers the opportunity to achieve desired bio-physicochemical features (including better efficacy and overcoming drug resistance). In this respect, *in silico* bio-pharmacokinetic characteristics were performed.

## 2. Materials and methods

### 2.1. Synthesis

All solvents and substances were purchased from Sigma-Aldrich and used without further purification.

To obtain (*R*)-(2-*tert*-butoxycarbonyl)amino-1-oxo-3-phenyl)propyl)-1-cyclopentene (**1**), a nucleophilic addition reaction was carried out at the carbon atom of the carbonyl group of a Weinreb amide, obtained in a known manner from *N*-(*tert*-butoxycarbonyl)-d-phenylalanine (Boc-d-Phe-OH) and *N*,*O*-dimethylhydroxylamine hydrochloride using any coupling reagent used in peptide synthesis. The use of *n*-butyl lithium (*n*-BuLi) instead of the more dangerous *sec*-butyl lithium to generate an anion on the sp^2^ carbon atom of the cyclopentenyl ring in cyclopentenyl iodide and the reaction of the obtained lithium derivative of cyclopentene with Boc-d-Phe-N(Me)OMe gave the expected ketone in moderate yield. More specifically, to a solution of 1-iodocyclopentene (6.63 g, 0.034 mol) in dry tetrahydrofuran (THF) (250 ml) was added *n*-butyl lithium (*n*-BuLi, 1 M solution, 70 ml, 0.070 mol) at −40°C. The mixture was stirred for 6 h to produce cyclopentenyl lithium, and cooled to −50°C, and then a solution of Boc-d-Phe-N(Me)(OMe) 7.7 g (0.025 mol) in 20 ml of anhydrous THF was added dropwise. The mixture was stirred for 3 h at −40°C and then overnight at room temperature. The reaction was then quenched with saturated ammonium chloride solution (150 ml), diluted with ethyl acetate (500 ml), and washed with 1 M KHSO_4_ (2 × 100 ml), 1 M KHCO_3_ (3 × 100 ml) and saturated NaCl solution (100 ml). After drying the organic phase over anhydrous MgSO_4_ and filtering off the drying agent, the solution was concentrated to obtain an oily residue, which was then diluted with hexane. After 24 h, the precipitate was filtered off, washed with hexane, and dried to give the desired ketone (4.01 g, 0.013 mol, 52% yield) as a cream-coloured powder of sufficient purity for use in subsequent syntheses. After precipitation of the product and concentration, the filtrate gave the unreacted Weinreb amide (3.1 g, 10 mmol).

### 2.2. X-ray single-crystal diffraction

#### 2.2.1 Crystal growth development

Suitable single crystals of **1** were grown via antisolvent vapour diffusion into the solvent. In particular, a beaker containing a solution of crystallized (*R*)-(2-*tert*-butoxycarbonyl)amino-1-oxo-3-(phenyl)propyl)-1-cyclopentene in methanol (antisolvent) was placed in a tightly closed vessel containing hexane (solvent). The vapours of the external solvent moved inside the antisolvent system by vapour diffusion, the solubility changed, and as a result, crystals were formed.

#### 2.2.2. X-ray structure determination

X-ray single-crystal diffraction data were collected on an XtaLAB Synergy Dualflex Pilatus 300 K diffractometer (Rigaku Corporation, Tokyo, Japan) equipped with a CCD type area detector using graphite monochromated Mo Kα (*λ* = 0.71073 Å) radiation, and processed using the Olex2 software [37]. The structure was solved with SHELXT [38] using CrysAlis PRO for data reduction [39] and refined with SHELXL97 using least-squares minimization. The non-hydrogen atoms were refined anisotropically. The hydrogen atoms were geometrically positioned and refined using a riding model.

Crystallographic data for **1** have been deposited at the Cambridge Crystallographic Data Centre with accession number 23 50 172 (refined re-submission), refrence code VORXIN [36]. Copies can be obtained, free of charge, on application to CCDC, 12 Union Road, Cambridge CB2 1EZ, UK (fax: +44 (0)1223 3 36 033 or Advancing Structural Science | CCDC (cam.ac.uk)).

Molecular diagrams and geometrical analysis were obtained using Mercury [40] and PLATON [41] programs.

### 2.3. Computational methodology

#### 2.3.1. Quantum-chemical calculations

**Density functional theory calculations.** Geometry optimization of a series of the neutral molecules under study starting from experimental structures in singlet ground states was performed using Gaussian 09 software [42]. The hybrid functional [43] with GD3 dispersion correction [44] and the basis sets from the Gaussian library [42] were used for this purpose. The optimized geometries were tested on the absence of imaginary vibrations.

**Quantum theory of atoms-in-molecules analysis.** Bonding interactions in molecules and crystals can be studied in terms of the QTAIM analysis of electron density [45]. Gradients of the electron density are equal to zero at its critical points, such as maxima, minima, and saddle points. Atom positions correspond to electron density maxima. Atom pairs can be linked by a line or a bond path along which the electron density is maximally concentrated. The bond path between two atoms is necessary for their chemical bonding. A molecular graph is a set of bond paths in a molecule or crystal. A bond critical point (BCP) is the saddle point at the bond path, and its characteristics are important for bond descriptors. The BCP electron density, *ρ*_BCP_, reflects the bond strength, its Laplacian, ∇^2^ *ρ*_BCP_, describes the relative electron density contribution of the bonded atoms (negative values correspond to covalent bonds):


$$\nabla^{2}\rho_{\text{BCP}}=\lambda_{1}+\lambda_{2}+\lambda_{3},$$

where *λ*_1_ < *λ*_2_ < 0 < *λ*_3_ are the eigenvalues of the Hessian of the BCP electron density. BCP bond ellipticity, *ε*_BCP_, describes its deviation from cylindrical symmetry (such as in ideal single or triple bonds) due to its double-bond character, mechanical strain, and other perturbations:


$$\varepsilon_{\text{BCP}}=\lambda_{1}/\lambda_{2}-1.$$

The bond energy *E*_b_ of weak interactions can be estimated as


$$E_{\text{b}}=\frac{1}{2}V(r_{\text{BCP}}),$$

where *V*(*r*_BCP_) is the potential energy density at BCP with the proportionality factor ½ being in volume atomic units [46].

Ring critical points (RCPs) are saddle points of electron density inside ring structures formed by bond paths. Local minima of the electron density are denoted cage critical points (CCPs). Nevertheless, the results of the QTAIM analysis must be treated carefully. Bond paths and BCPs correspond to conventional chemical bonds in most cases, but not all. There is no universal correspondence. QTAIM analysis was performed using AIMAll software [47] from .wfn files produced by Gaussian 09 software. Molecular graphs were drawn with AIM2000 software [48].

#### 2.3.2. Docking protocol

**Molecular docking preparation**. Molecular docking for **1** was performed using AutoDock Vina v. 1.1.2 (http://autodock.scripps.edu/) [49]. First, ligand **1** in non-ionizable form was prepared with ChemAxon MarvinSketch v. 14.9.1.0 (http://www.chemaxon.com/marvin/). Next, the initial geometries of the ligand with the minimum energy conformation were optimized in Avogadro v. 1.2.0. (http://avogadro.cc/) using MMFF94 force field with 500 steps and the Steepest Descent Algorithm. Afterwards, the Gasteiger partial charges for **1** were calculated with AutoDock Tools v. 1.5.6 (ADT, S3; http://mgltools.scripps.edu/). In contrast, all torsion angles for (*R*)−**1** were considered flexible, and all the possible rotatable bonds and nonpolar hydrogens were determined. The final ‘ligand’ file appropriate for docking calculations was saved in PDBQT format (.pdbqt). The crystal structures of human protein kinases, namely CK2**-**α (PDB code: 4KWP) [50] of the highest available resolution (1.25 Å), and PIM-1 (PDB code: 4DTK) [51] with resolution 1.86 Å, were downloaded from the PDB database (http://www.rcsb.org/pdb/). The crude target proteins were prepared by means of the UCSF Chimera v. 1.11.2 package (http://www.cgl.ucsf.edu/chimera/) [52] after removing all nonstandard molecules, including 4,5,6,7-tetrabromo-1-(2-deoxy-β-d-erythro-pentofuranosyl)-1*H*-benzimidazole (EXX), 1,2-ethanediol (EDO), dimethyl sulfoxide (DMS), triethylene glycol (PGE), sulfate ion (SO_4_) and di(hydroxyethyl)ether (PEG) in the case of CK2**-**α (PDB code: 4KWP) as well as (5*Z*)-5-{2-[(3*R*)-3-aminopiperidin-1-yl]-3-(propan-2-yloxy)benzylidene}-1,3-thiazolidine-2,4-dione (7LI), EDO and SO_4_ in the case of PIM-1 (PDB code: 4DTK).

**Molecular docking procedure.** Docking was carried out on a 24 CPUs-based desktop computer equipped with AMD Ryzen™ 93900X12 Core Processor 3800 MHz and 32 GB of RAM on a Microsoft Windows 11 Professional 64-bit operating system using a standard protocol as described previously [53]. Briefly, docking was performed using the advanced protein flexibility option by specifying flexible sidechains. Each docking was calculated with an exhaustiveness level of 96 concerning global search. For each ligand molecule, 100 independent runs were executed using the Lamarckian Genetic Algorithm (GA) with at most 106 energy evaluations and a maximum number of generations of > 27 000 Å^3^ (the search space volume). The rest of the docking parameters, including the remaining Lamarckian GA parameters, were defaulted using the standard values for genetic Vina algorithms (the posed dockings were below 5.00 Å root mean square deviation (r.m.s.d.). A searching ‘grid box’ was set by using the AutoGrid function to perform docking in a (40 × 40 × 40 Å)-unit grid box (with a final size space dimension of *x* = 40 Å, *y* = 40 Å, *z* = 40 Å), centred on catalytic cavity located in CK2**-**α (centre_*x* = 21.580; centre_*y* = −31.180; centre_*z* = 13.278) or ATP-binding site of the PIM-1 (centre_*x* = 18.633; centre_*y* = −35.091; centre_*z* = −1.101) as target coordinates with a grid spacing of 0.325 Å, respectively. Silmitasertib (CX-4945) was docked as a control ligand to validate the docking calculations. The docking modes for both the studied ligands, **1** and CX-4945, were clustered and ranked based on a mutual ligand–protein affinity expressed as absolute free binding energies, Δ*G*_calc_ (kcal mol^−1^; 1 kcal = 4.184 kJ), as well as the values of r.m.s.d. in both modes regarding r.m.s.d. lower bound (l.b.), and r.m.s.d. upper bound (u.b.), respectively. The r.m.s.d. values were computed for the input structure submitted to docking simulations. For CK2**-**α (PDB code: 4KWP), the used random seed amounted to +19 79 76 760 for (*R*)−**1** and −89 90 95 056 for CX-4945, whereas for PIM-1 (PDB code: 4DTK), the used random seed amounted to −45 75 16 032 for (*R*)−**1** and +17 50 572 488 for CX-4945, respectively. The best nine poses (binding modes) were selected according to AutoDock Vina scoring functions, which were mainly based on binding energies and showed mutual ligand–protein affinity (kcal mol^−1^). The optimized binding poses of **1** and CX-4945 in hypothetical complexes with CK2**-**α and PIM-1 were visualized using PyMOL v. 1.3 molecular graphics system software (Schrödinger; https://www.pymol.org/).

#### 2.3.3. Hirshfeld surface analysis

Three-dimensional HS maps and corresponding two-dimensional fingerprint plots were obtained using CrystalExplorer 21.5 software [54–56]. The mapping of interactions on HSs was interpreted as short/long contacts in the crystal lattice. Interactions on the *d*_norm_ maps are represented by colour-coded surfaces: red for shorter, blue for intermediate, and white for longer contacts than the sum of van der Waals (vdW) radii of atoms. Here, *d*_norm_ is the normalized contact distance, which is based on the distances from the nearest atom inside (*d*_i_) and that outside (*d*_e_) the HS to the relative to the vdW radiius of the atom. We have also analysed additional properties, such as the shape index and curvedness, based on the local curvature of the surface as well as the fragment patch. Two-dimensional fingerprint plots were determined based on intermolecular interaction information via summation of *d*_e_ and *d*_i_ in the crystal lattice obtained from the three-dimensional HS [57,58]. Histograms provide the relative area of the surface related to all kinds of interaction present in the crystal. The percentage contribution of diverse contact pair was obtained from these plots.

The *enrichment ratios* were calculated based on the contact surface’s decomposition values between interacting pairs. The likelihood of privileged and disfavoured interactions in the stabilization of the crystal packing was analysed [58].

*Interaction energies and energy frameworks* were calculated for all unique molecular pairs within the 3.8 Å radius range of the first coordination sphere of a molecule using the CrystalExplorer program. The electron density of the molecules has been obtained at the B3LYP/6-311G (d,p) level of theory [54]. The total interaction energy was partitioned into Coulombic, polarization, dispersion, and repulsion energy contributions. The energy threshold was kept at 0.00 kJ mol^−1^ and tube size at 100.

#### 2.3.4. ADME-T and beyond

The ADME-T (absorption, distribution, metabolism, excretion and toxicity) characteristics of analysed compounds were obtained via *in silico* calculations using SwissADME (Molecular Modelling Group of the Swiss Institute of Bioinformatics) [59,60] as well as pkCSM interface [61]. The structures were converted into canonical simplified molecular input line entry specifications (SMILES). In addition, the bioactivity scores were calculated on the platform Molinspiration Cheminformatics (https://www.molinspiration.com). The tumour (and non-tumour) cell line cytotoxicity was obtained by the CLC-Pred tool based on structure–cell line cytotoxicity relationships using the PASS procedure (activity spectra for substances) [62].

### 2.4. Cambridge Structural Database survey

A search of the CSD, v. 5.43, update of November 2023 [63], confirmed the novelty of **1**. A screen of the most similar cyclopentene-containing peptide-based compounds that can form intramolecular interactions gave five relevant hits, with the following specifications: good quality, no disorder, no polymeric, no powder structures, only organics, no errors, no duplicates and complete three-dimensional coordinates. The CSD reference codes are as follows: EBIMUY [64], RUCBEZ [65], BECNUV [66], OKEWEG [67] and TOBSUA [68] (table 1). Nevertheless, only EBIMUY [61] shows a similar pseudo-bicyclic system. Therefore, despite the fact that it contains another bioisosteric moiety—an imidazoline ring, which is also similar to cyclopentene—it was included in the analysis. A list of the retrieved crystal structures, containing basic crystallographic data, is provided in table 1. The molecular views of the structures are presented in electronic supplementary material, figure S1.

**Table 1.** Crystal data for analysed compounds. v-volume, r-r factor = theses obvious for crystallographers.

	formula	space group	unit cell parameters (Å, ^o^)	*V* (Å^3^)	*R*
1*	C_19_H_25_NO_3_	*P*2_1_2_1_2_1_	*a* = 10.6291(4) *b* = 18.0980(7) *c* = 18.7900(7) α = *β* = *γ* = 90^o^	3614.547	0.0345
EBIMUY	C_24_H_28_N_4_O_5_, 0.25 (C_4_H_10_O)	*P*-1	*a* = 10.754(1) *b* = 14.735(1) *c* = 17.497(1) *α* = 112.79(1)^o^ *β* = 91.02(1)^o^ *γ* = 97.03(1)^o^	2530.328	5.45 (room temp.)
diethyl *N*-(1-benzyl-1-(2-imidazolin-2-yl)-2-phenyl-2-oxoethyl)hydrazine-*N*,*N*'-dicarboxylate diethyl ether solvated					
RUCBEZ	C_26_H_29_N_3_O_6_S	*P*2_1_/n	*a* = 10.089(1) *b* = 23.696(1) *c* = 11.176(1) *α* = γ = 90.00^o^ *β* = 91.1891^o^	2671.339	5.88 (room temp.)
diethyl 1-{(cyclopent-1-en-1-yl)[1-(phenylsulfonyl)-1*H*-indol-3-yl]methyl}hydrazine-1,2-dicarboxylate					
BECNUV	C_12_H_12_INO	*P*-1	*a* = 7.605(1) *b* = 11.856(1) *c* = 12.819(1) *α* = 88.13(1)^o^ *β* = 85.25(1)^o^ *γ* = 87.59(1)^o^	1150.534	2.58 (100 K)
*N*-(2-iodophenyl)cyclopent-1-ene-1-carboxamide					
OKEWEG	C_16_H_28_N_2_O_6_SSi	*P2*_*1*_ *2*_*1*_ *2*_*1*_	*a = 6.334(2)* *b = 10.812(4)* *c = 31.745(12)* *α = β = γ = 90* ^*o*^	2173.999	11.19 (150 K)
3-butoxycarbonyl-4-(2-(trimethylsilyl)ethylsulfonamido)cyclopentene[*c*][1,3]oxazolidin-2-one					
TOBSUA	C_15_H_18_N_2_OS	*P*-1	*a* = 6.950(1) *b* = 9.462(1) *c* = 11.326(1) *α* = 71.52(1)^o^ *β* = 81.83(1)^o^ *γ* = 89.24(1)^o^	698.801	4.13 (room temp.)
*N*-((2-phenylethyl)carbamothioyl)cyclopent−1-ene-1-carboxamide					

Other key crystal data for **1**: *M*_r_ = 315.40, *Z* = 8, *ρ* = 1.159 g cm^−3^, *μ*(Mo-Kα) = 0.078 mm^−1^, reflections: 4843 collected, 4332 unique, *R*_int_ = 0.0231, *R*_1_ (all) = 0.0345, w*R*_2_ (all) = 0.1027, *S* = 1.088.

## 3. Results and discussion

### 3.1. Structural description and supramolecular analysis

**Figure 1.**
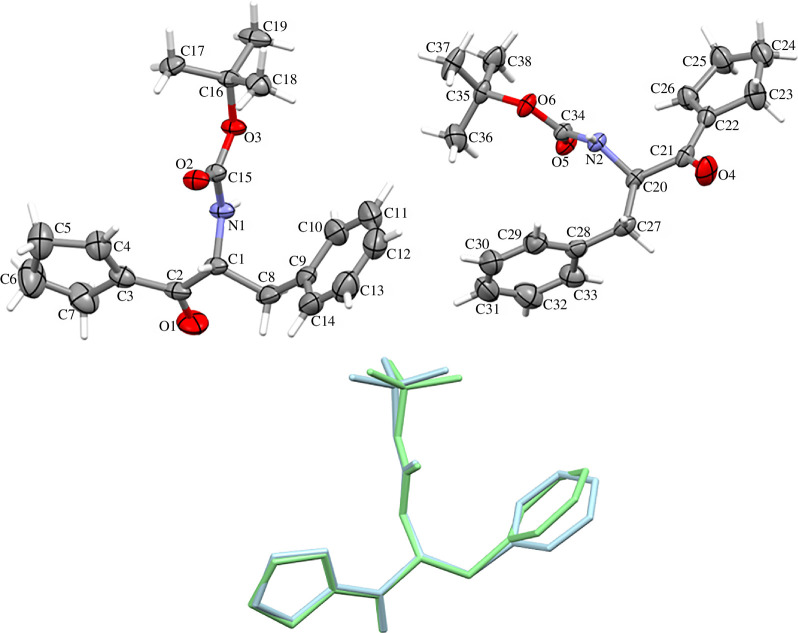
Above: the asymmetric unit of **1**, showing 50% probability thermal ellipsoids. Below: overlay of molecules.

Novel compound (*R*)-(2-*tert*-butoxycarbonyl)amino-1-oxo-3-phenyl)propyl)-1-cyclopentene (**1**) crystallizes in an orthorhombic crystal system with a *P*2_1_2_1_2_1_ space group with two independent molecules with a similar conformation in the asymmetric unit (figure 1). The molecules have *R* chiral centres at C1 and C20 atoms. The phenyl rings are planar. One of the cyclopentene rings (C22–C23–C24–C25–C26) adopts a slightly flattened envelope conformation with average puckering parameters *Q* = 0.103(3) Å, *ϕ* = 251.1(16)^o^ [69]. The cyclopentenes lie perpendicularly towards the amide plane. The amide bond is in a *trans* conformation. Of note, several important details were observed from X-ray data. In particular, the X-ray structure of **1** shows a network of H-bonds involving both molecules.

The intramolecular C–H^…^O as well as intermolecular N–H^…^O and C–H^…^O H-bonding interactions ((in the range 2.21(2) to 2.54(4) Å)) have relevance in establishing the organization of the extended supramolecular structure, which can be seen in electronic supplementary material, figure S2. More specifically, hydrogen-bonded self-assembled supramolecular units generate a zig-zag chain-like structure, forming intermolecular interactions between amine/alkyl and carbonyl groups. To be more precise, nitrogen atoms of amine groups in both molecules act as donors participating in the formation of N1–H2^…^O5 (1*x*, *−*1/2 ½ *+ y,* ½ *− z*) and N2–H1^…^O2 (½ *+ x*, ½ − *y*, *–z*); hence, supramolecular chains and distinct motifs (described below) are formed. On the other hand, nearly all oxygen atoms of carbonyl groups play the role of acceptors participating in the generation of either the same N–H^…^O interactions or C–H^…^O interactions, leading to the corresponding H-bonding supramolecular motifs. The interplay of these H-bonding motifs results in the three-dimensional network formation. The structure is stabilized by π-based interactions. The geometric parameters of hydrogen bonds are summarized in table 2 for **1** and in electronic supplementary material, table S1, for other analysed compounds retrieved from the CSD. Thus, novel structures can be used in crystal engineering owing to the possibility of the formation of diverse supramolecular synthons [70–72] at the subsequent levels of supramolecular architecture. Both molecules establish intramolecular hydrogen bonds C–H^…^O between the carbonyl groups and cyclopentene ring, forming an *S*(8) supramolecular motif in the graph-set assignments. Interestingly, it is stabilized via C–O^…^π contact between the carbonyl group and the phenyl ring. In consequence, the cyclopentene-based pseudo-bicyclic system shielding the amide bond has been observed for the first time to the best of our knowledge. We realized that cyclopentene can encode molecular ‘chameleonicity’ by shielding polarity via intramolecular interactions (and potentially enhancing permeability). This information could be utilized in designing peptide-based molecular chameleons. More specifically, at the first level of graph theory [73,74], apart from the above-mentioned S(8) motif (via the C4–H4^…^O2 and C26–H26^…^O5 interactions in both molecules, respectively), D(2) synthon (by the N1–H2^…^O5/N2–H1^…^O2) and C(9) (via C19–H19B^…^O1/C37–H37^…^O4 interactions) are observed. At the second level, the same types of motifs are created for the other molecule (R)-(2-*tert*-butoxycarbonyl)amino-1-oxo-3-phenyl)propyl)-1-cyclopentene, and, among others, eight- and nine-membered linear motifs C^2^_2_(8) and D^3^_3_(14) for both molecules in the crystal with the participation of N1–H2^…^O5 (½ *+ x,* ½ *− y, −z*)/N2–H1^…^O2 (½ *+ x,* ½ *– y, –z*), N1–H2^…^O5 (½ *+ x*, ½ *− y, −z*) and C37–H37B^…^O4/N2–H1^…^O2 (½ *+ x,* ½ *− y, −z*) and C19–H19B^…^O1/N2–H1^…^O2 (½ *+ x,* ½ *− y, −z*) and C37–H37B^…^O4, which are realized in the mode of the repeated patterns of fused rings (see electronic supplementary material, figure S2). * intramolecular interactions.

**Table 2.** Geometrical parameters of hydrogen bonds in **1**. * intramolecular interactions.

D–H^…^A	*d* (H^…^A) (Å)	*d* (D^…^A) (Å)	(D–H^…^A) (^o^)
**1**			
N–H1^…^O2^a^	2.27(2)	3.0728(17)	157.5(19)
N1–H2^…^O5^b^	2.21(2)	3.0683(18)	164.3(18)
C37–H371^…^O4^c^	2.44(3)	3.321(3)	151(2)
*C38–H8^…^O5	2.43(3)	2.999(3)	113(2)
*C1–H11^…^O2	2.45(2)	2.8353(19)	103.4(14)
*C17–H173^…^O2	2.48(3)	3.019(2)	115(2)
*C18–H181^…^O2	2.42(3)	2.959(3)	115(2)
*C20–H201^…^O5	2.404(19)	2.8284(19)	105.8(13)
*C36–H361^…^O5	2.54(4)	3.059(4)	114(3)

*^a^* ½ *+ x,* ½ *− y, −z*; ^b^1*x, −*½*+ y,* ½ − *z*; ^c^ *−*1 *+ x, y, z.*

**Figure 2.**
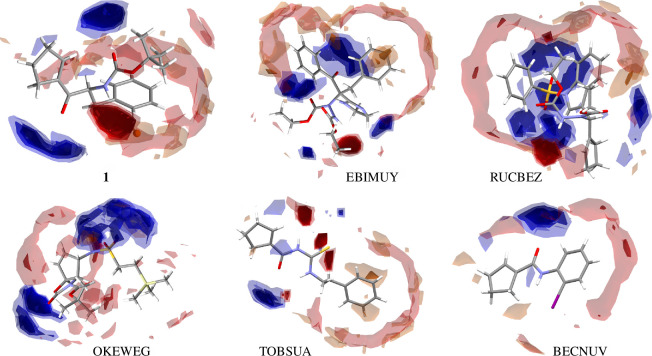
Full interaction maps for analysed compounds. Blue and red areas signify the most probable locations of hydrogen bond donors and acceptors, respectively. Orange and brown surfaces denote the preferences for aromatic interactions.

**Figure 3.**
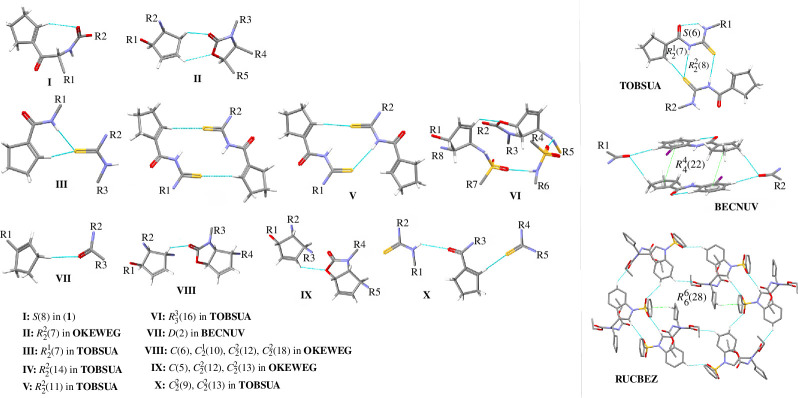
On the left: library of cyclopentene-based H-bonding motifs in analysed peptide-derived structures. On the right: specific features of selected motifs such as triple-synthon or stabilization of motifs via cyclopentene-based π^…^π or C–H^…^π contacts.

**Figure 4.**
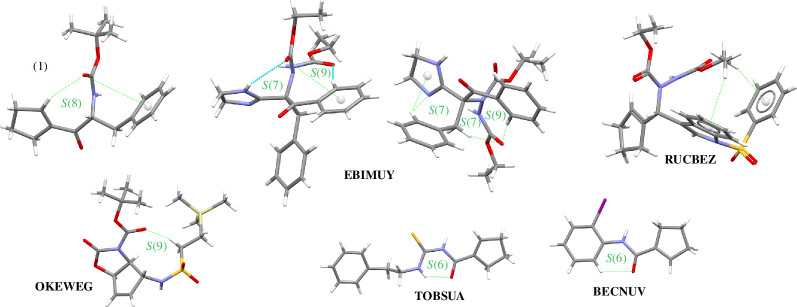
Pseudo-cyclic motifs via weak noncovalent interactions observed in analysed crystals.

To gain deeper insight into the supramolecular landscape of cyclopentene-containing peptide-based structures, we extended the analysis to other crystal structures, such as EBIMUY [61], RUCBEZ [62], BECNUV [63], OKEWEG [64], TOBSUA [65], that are similar to those that can form intramolecular motifs. The crystal data of all analysed structures are shown in table 1. The geometrical parameters of H-bonds are included in table 2, while the remaining data concerning π-based interactions are summarized in electronic supplementary material, table S2. Diverse substituents in the analysed structures interfere with supramolecular motifs via weaker interactions. Therefore, the typessupramolecular and motifs generated by these interactions are worth exploring. The suitable complementary co-formers were found using full interaction maps (FIMs) [75]. These are a suitable data-driven tool for either analysis or visualization of the spatial arrangement of functional groups (in the analysed molecules: the amide, carbonyl, secondary amide atoms, cyclopentene, (iodo)phenyl and imidazoline ring, *tert*-butyl, sulfonyl silyl and thiol group) that form intermolecular interactions in an effective way. Three-dimensional maps (figure 2) were calculated based on all structures deposited in the CSD, including diverse structural factors (*inter alia* steric repulsions) [33]. Thus, a FIM provides statistical information about not only possible H-bonds but also possible π-based intercontacts of selected molecules by probing the entries of the CSD. The most populated places are the most likely donors and acceptors (blue and red areas, respectively). In the structure of **1**, the O2 and O5 atoms are the most highly populated accessible H-bond acceptors, which is in line with electrostatic potential (EP) maps (described and presented in the following sections). An area for donors is near N1 and N2 atoms. The above-mentioned H-bonding interactions are well related to the maps. In addition, beige-brown surfaces pointing to the aromatic intercontacts are visible. In maps for OKEWEG and TOBSUA, we can see the most cyclopentene-based interactions. In particular, in the majority of compounds, C–H^…^O is observed, and also in TOBSUA, C–H^…^S, while in BECNUV (and TOBSUA), π^…^π intercontacts. Notably, cyclopentene plays the role of either acceptor or donor and forms basic synthons in all crystals (apart from RUCBEZ). An incisive analysis of hydrogen-bonded patterns and their three-dimensional crystal packing networks in peptide-derived crystals has revealed several distinctive supramolecular motifs. Noteworthy, cyclopentene forms all kinds of synthons (S, R, C, D) via O(N,C)-H^…^O and N(C)-H^…^S interactions (electronic supplementary material, table S3, figure 3). In addition, it can be mentioned that only in TOBSUA and RUCBEZ is the centrosymmetric dimeric motif, homosynthon R^2^_2_(8) via N–H^…^S and N–H^…^O interactions, respectively, observed. This motif can form a dual/triple synthon. Notably, cyclopentene participates in the stabilization of motifs via C–H^…^π and also π^…^π intercontacts (as an electrostatic compression effect). From figure 4, it can be seen that cyclopentene in **1** is directly engaged in shielding the amide bond by forming a pseudo-cycle system, which increases the value of the novel compound and sheds light on a new perspective in designing ‘molecular chameleons’. Besides, the amide bond is shielded by intramolecular C–H^…^O (in **1**, EBIMUY, OKEWEG, BECNUV), N–H^…^O (in EBIMUY, TOBSUA), C–O^…^π (in **1**, EBIMUY) and C–O^…^π (in RUCBEZ) interactions. In TOBSUA and BECNUV, intramolecular H-bonding motifs, denoted S(6), are equivalent (interchangeable) synthons. They can be employed in designing new molecules in the context of a ‘pseudo-ring replacement strategy’. A library of all observed H-bonding motifs is included in electronic supplementary material, table S3.

### 3.2. Long*-*range synthon *Aufbau* modules

**Figure 5.**
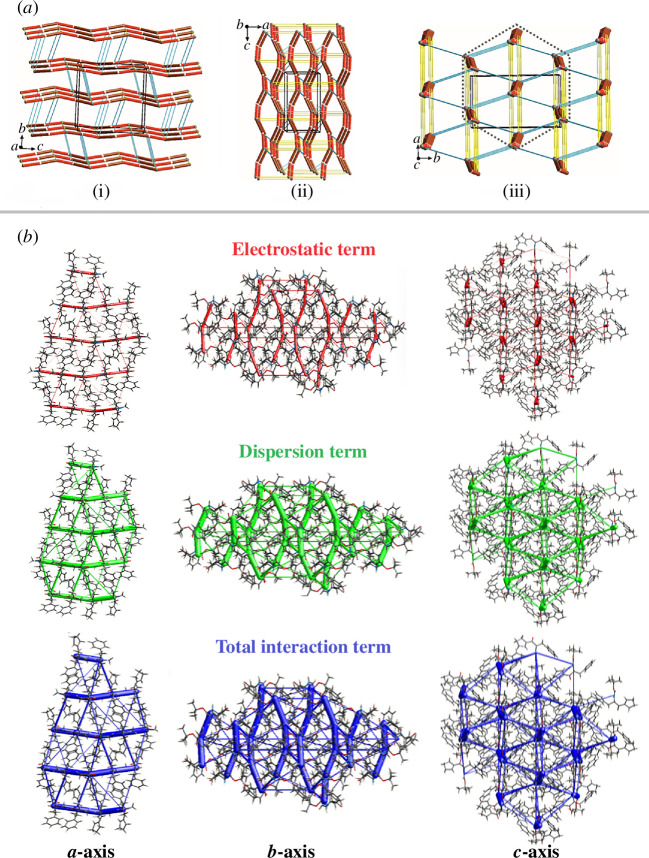
(*a*) Topology of the network formed by the most important intermolecular interactions in **1**. The molecules A and B are represented by their gravity centres (white and grey balls). The N–H^…^O hydrogen bonds are shown as red lines, whereas weaker C–H^…^π and C–H^…^O contacts are denoted as blue and yellow thin lines, respectively. ( (i)–(iii)) Views along the appropriate crystal lattice basis vectors. The dotted hexagon highlights the packing of one-dimensional long-range synthon *Aufbau* modules (LSAMs). (*b*) Perspective views of the energy frameworks(EF) calculation (electrostatic, dispersion and total interaction energies) for a cluster of nearest-neighbour molecules in **1** (the energy tube size is 100, and the energy threshold is 0 kJ mol^−1^).

The molecules **1**, EBIMUY and RUCBEZ have some features in common (apart from a tendency to form similar intramolecular systems, a similar share of O^…^H and C^…^H, and a minor proportion of N^…^H interactions in HSs), whereas the remaining structures, because of a greater diversity of interactions and significant contribution of I–H, S–H interactions in the supramolecular organization, differ sharply. Therefore, we consider long-range synthon *Aufbau* modules (LSAMs) only in **1**, EBIMUY and RUCBEZ. Two crystallographically independent **1** molecules (A and B) form an infinite chain structure through N–H^…^O hydrogen bonds of estimated energy *ca* 70 kJ mol^−1^ (see the results of the Crystal Explorer calculations). The chains comprise alternately connected molecules A and B and spread along the [001] direction. The one-dimensional structure is strengthened by aromatic interactions between almost parallel-oriented phenyl rings of subsequent molecules. These interactions were classified as strong with a rating of 8.2 according to the Aromatic Analyzer module integrated with the CSD-Materials package [40]. This one-dimensional structure can be identified as an LSAM [76,77], taking into account that the other identified intermolecular interactions, C–H^…^π and C–H^…^O, have significantly lower energy of around 20 and 10 kJ mol^−1^. These weaker interactions join the supramolecular chains into the three-dimensional structure. Their secondary role is disclosed when analysing the topology of intermolecular connection. In figure 5, the molecules of **1** are reduced to points, represented by their gravity centres, while the identified intermolecular interactions are shown as lines (thick lines denote N–H^…^O hydrogen bonds, blue and yellow thin lines stand for C–H^…^π and C–H^…^O contacts, respectively). A view down the *c*-axis, along the main supramolecular motif, makes it clear that the one-dimensional chains are almost hexagonally packed. Hence, the identified weaker interactions are only partially directional.

A short discussion of LSAMs in EBIMUY and RUCBEZ is presented in electronic supplementary material, figure S3.

### 3.3. Hirshfeld surface analysis

**Figure 6.**
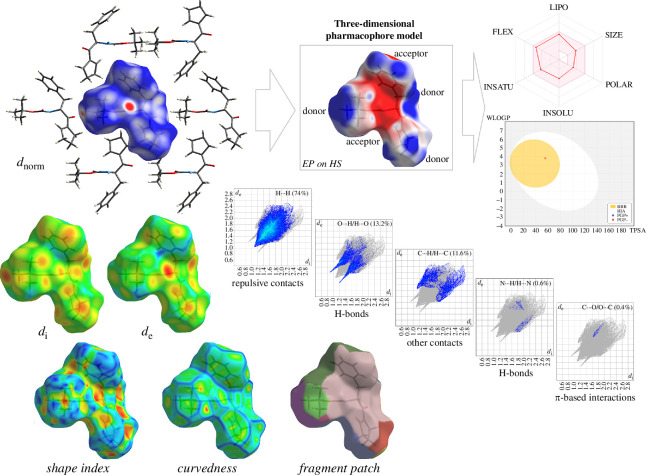
From left to right: Hirshfeld surfaces (HSs) mapped with *d*_norm_, *d*_i_, *d*_e_, shape index, curvedness and fragment patch; fingerprint plots delineating H^…^H, O(C,N)^…^H/H^…^O(CN), C^…^O/O^…^C interactions; electrostatic potential (EP) mapped on the HS (EP on HS) presented as three-dimensional pharmacophore model; Bioavailability Radar and BOILED-Egg plots for **1**. lipo - lipophilicity, size - molec. weight, polar - polarity, insolu - insolubility, insatu - insaturation, flex - flexibility.

**Figure 7.**
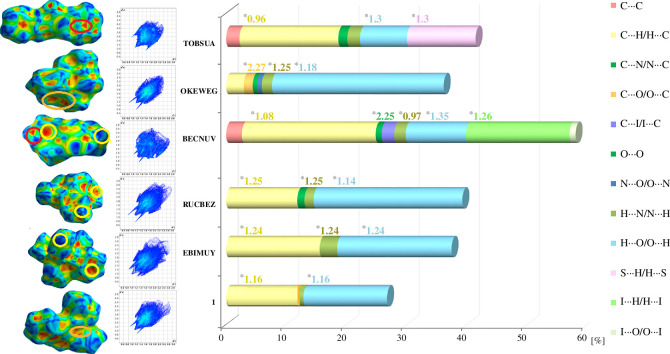
From left to right: illustration of corresponding interactions on the Hirshfeld surfaces (HSs) (mapped over the shape index property in the colour range of −1.0 to 1.0), full fingerprint plots, and the relative percentage contribution of individual intermolecular contacts (above 0.5%), without H^…^H, to the HS area of the analysed crystals (* denotes enrichment ratio values).

We applied HS analysis [54,56,57,78] to better understand the contribution of especially weak intermolecular interactions to the crystal packing of all crystals. The HSs and their fingerprint plots for **1** are illustrated through *d*_norm_ (−0.1 to 1.5 Å), *d*_i_, *d*_e_, shape index, curvedness and fragment patch in figure 6, and for other compounds in figure 7 and electronic supplementary material, figure S4, respectively. The HS map plotted as *d*_norm_ presents diverse red areas, equivalent to the above-considered intermolecular interactions. On the *d*_norm_ map, the strongest large red circular impression chiefly discloses N–H^…^O, while a smaller spot is attributed to C–H^…^O interactions. In addition, acceptor and donor locations are revealed from the HS mapped as *d*_e_, *d*_i_. On the shape index map, C–O^…^π contact is shown (figure 7). Overall, this type of map is representative of C^…^C (π^…^π) contacts. The characteristic pattern of adjacent red and blue triangles is visible on the map for BECNUV and TOBSUA (figure 7) but not for **1** (figure 6). To shed deeper light on π^…^π interactions, the molecular surfaces of these crystals demonstrated over curvedness contain characteristic broad flat green regions over the aromatic rings with dark blue edges (see electronic supplementary material, figure S4). The largest region of flat curvedness appears for BECNUV. It is in agreement with the crystallographic data and simplifies the distribution of weak contacts, giving rise to a hierarchical list of them as well as the interpretation of packing motifs. The existence of the C–H^…^π interactions is evidenced *inter alia* by the appearance of patches with large red concave regions above the π-system and blue bumps as convex areas surrounding the C–H donor on the HSs mapped over the shape index on the plots for EBIMUY, RUCBEZ and BECNUV. Nevertheless, it should be mentioned that C–H^…^π interactions are also visible over the *d*_e_ property on the HS maps.

Finally, the collection of colour patches specifies the number of the nearest surrounding molecules.

The molecular EP applied to the HS illustrates the electrostatic reactivity of **1**. Electronegative (red) and electropositive (blue) regions characterize the acceptor and donor sites, respectively (figure 6), constituting a three-dimensional map of the pharmacophore. Though they look visually identical, all EPs are different, and the dipolar character of the EBIMUY map original.

Using diverse properties of HS maps and the breakdown of fingerprint plot information, it is possible to graphically identify those areas of the surface that are involved in a specific type of intermolecular interaction. Here, two-dimensional fingerprint plots are used to present the contribution of individual close intercontacts in a novel visual manner (figures 6 and 7) [79]. Figure 6 demonstrates the decomposing fingerprints (FPs) of **1** in the crystal lattice, highlighting the corresponding intermolecular contacts with their percentage contributions (> 0.5%) separately. Dominant spikes, apart from H^…^H contacts (74%), appear for H^…^O/O^…^H and C^…^H/H^…^C, with a contribution of 13.2% and 11.6%, respectively (electronic supplementary material, table S4). The smallest distance (*d*_e_ *+d*_i_ ≈ 2.1 Å) is related to a pair of spikes for H^…^O/O^…^H contacts, while the longest distance (*ca* 3.1 Å) is related to a two broad spikes for C–H (11.2%). Notably, wings characteristic for C–H^…^π interactions are noticeable. Other close contacts such as N^…^H/H^…^N and C^…^O/O^…^C are also evident in the fingerprint plots of **1**, but their contribution is much lower (at the level of 0.5%).

The fingerprint plots and a histogram of the percentage contribution of main contacts for all six studied structures are shown in figure 7. It can be mentioned that a significant contribution comes from H^…^H contacts (from 42% in BECNUV to 74% in **1**), which is not surprising since hydrogen atoms constitute the outer ‘layer’, favouring the formation of close contacts. They can play the role of ‘supramolecular glue’ in the crystals. More importantly, the substituents play a key role in the participation of diverse types of contacts. The contribution of dominant O^…^H/H^…^O and C^…^H/H^…^C contacts is from 24.2% in TOBSUA to 36.5% in RUCBEZ. The greatest share of N^…^H/H^…^N interactions is at 2.9% in EBIMUY. C(π)^…^C(π) contacts have relevance only in BECNUV (2.6%) and TOBSUA (2.2%), C^…^O/O^…^C in OKEWEG (1.5%), and O^…^O in RUCBEZ (1.2%). This is followed by contributions from I^…^H/H^…^I, C^…^I/I^…^C and I^…^O/O^…^I in BECNUV; S^…^H/H^…^S in TOBSUA with a contribution of 17.2, 2.1 and 1%; 11.4%, respectively (figure 7; electronic supplementary material, table S4).

For deeper insight, we have also established that the reciprocal H^…^O/O^…^H and H^…^C/C^…^H are highly favoured in all crystals as well as H^…^N (apart from **1** and TOBSUA), H^…^S/S^…^H (in TOBSUA), C^…^O/O^…^C (in OKEWEG), H^…^I/I^…^H and C^…^N/N^…^C (in BECNUV), since their corresponding enrichment ratios are equal to or larger than unity; see figure 7 and electronic supplementary material, table S5. At this point, it should be recalled that the enrichment ratio is defined as the ratio of real interactions in the crystal to the calculated interactions with the same probability of formation [58]. Thus, the pairs of atoms with an enrichment ratio above unity exhibit a high tendency to form intercontacts. In other words, they are privileged in the crystal lattice. On the contrary, contacts with an enrichment ratio less than unity are much less favoured/impoverished. The results of calculations along with the actual and random contacts of the main intercontacts for all analysed compounds are collected together in electronic supplementary material, table S5. For example, most interactions in **1** are H···O and C^…^H type, and they are enriched, having an enrichment ratio of 1.16. This is due to the abundance of the corresponding contributions related to these interactions to the total HS area. Moving forward, among all types of contacts in all studied crystals, C^…^O interaction in OKEWEG and C^…^N in BECNUV exhibit the highest enrichment ratios: 2.27 and 2.25, acquiring only 1.5 and 1% surface area in the HS, respectively. Interestingly, C^…^C interactions are not preferable since the value of the enrichment ratio is below unity, despite those interactions contributing at a level of *ca* 2.5% in BECNUV and TOBSUA. Nevertheless, overall, as seen in figure 7, weak intercontacts are preferable in this class of compounds.

Further, EFs were calculated to provide an inclusive understanding of the interaction topology and an in-depth analysis of intercontact energies. More specifically, the contribution of electrostatic energy (*E*_ele_), polarization energy (*E*_pol_), dispersion energy (*E*_dis_) and repulsive energy (*E*_rep_) to the total energy (*E*_tot_) of the interactions was evaluated, where the molecular cluster is mapped within 3.8 Å. The EFs visualize the estimation of the intermolecular interaction energies of molecular pairs in the crystal lattice. They are illustrated by coloured rods connected by centroids of molecular pairs across the crystal using crystallographic symmetry operations. Thus, it can be noticed that EFs display hierarchical packing of LSAMs. The strength of resulting intermolecular interactions is determined to be related to the radius of the rods (cylindrical tubes). The tube size is adjusted with a scale factor of 100. The scale factors are adjusted to *K*_ele_ = 1.057, *K*_pol_ = 0.740, *K*_dis_ = 0.871, *K*_rep_ = 0.618, with a cut-off value of 5 kJ mol^−1^ [54]. EFs are presented as red, green and blue rods for *E*_ele_, *E*_dis_ and *E*_tot_, respectively (figure 5; electronic supplementary material S5). The calculated lattice energy parameters are tabulated in electronic supplementary material, table S6. The resultant calculations for compound **1** disclose different energy modules—in particular, −81.5 (*E*_ele_), −29.7 (*E*_pol_), −265.3 (*E*_dis_), 119.5 (*E*_rep_) and −244.3 kJ mol^−1^ (*E*_tot_). Among the generated components, the larger value of dispersion energy indicates dominant weak noncovalent interactions compared with hydrogen bonding interactions in terms of electrostatic forces in crystal packing. Thus, as we can see in figure 5, the size of the green cylinder in the dispersive force framework is larger than that of the red cylinder in the electrostatic force structure. Furthermore, we should highlight that the relationship of energy terms is analogous to other compounds.

### 3.4. Quantum-chemical calculations

**Figure 8.**
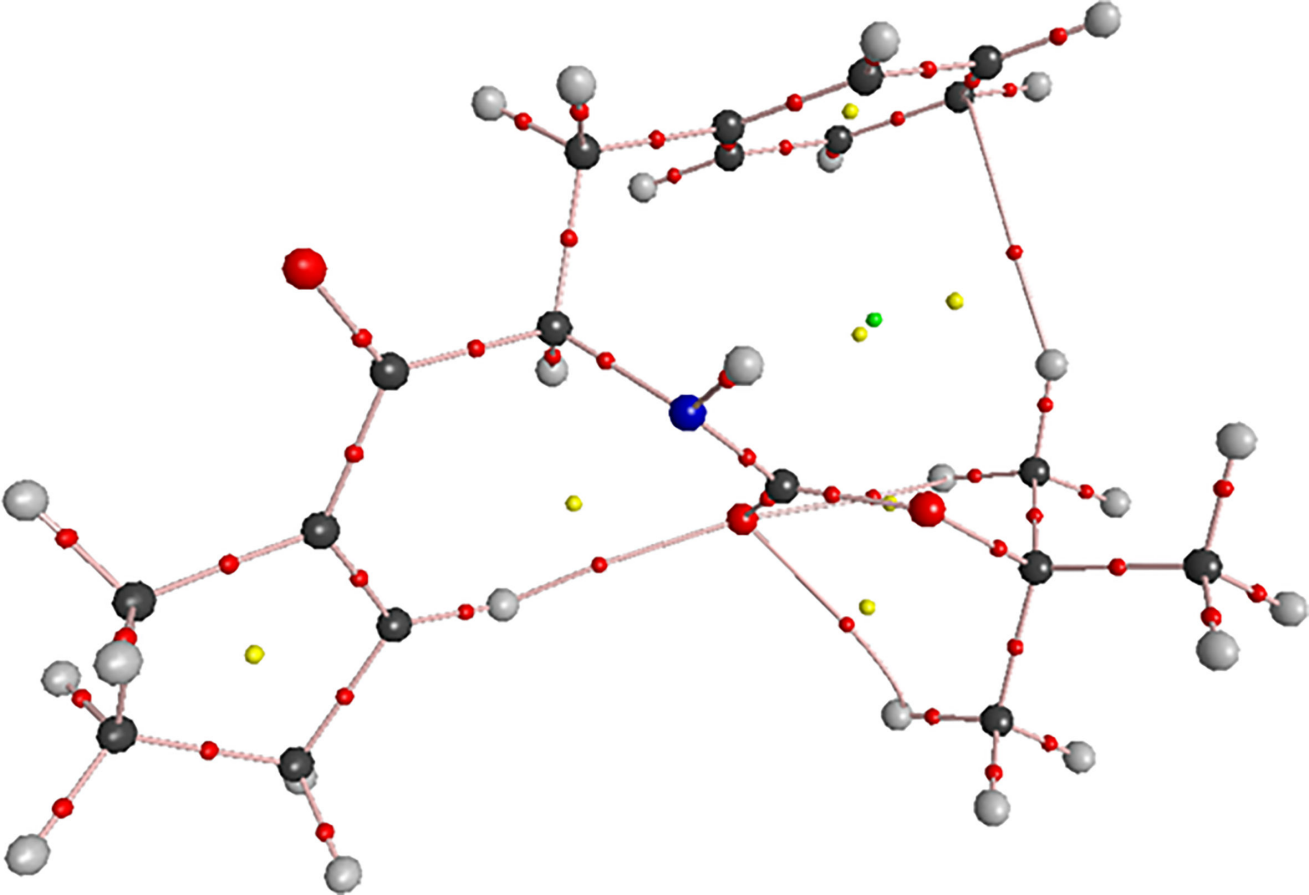
Molecular graph of **1** (C—black, H—grey, O—larger red, N—blue, bond critical point (BCP)—small red, ring critical point (RCP)—yellow).

We have investigated DFT-optimized structures of single molecules of **1**, EBIMUY, RUCBEZ, OKEWEG, TOBSUA and BECNUV for weak interactions in terms of QTAIM. Their molecular graphs are presented in figure 8 and electronic supplementary material, figures S6–S10. The weak interactions observed can be divided into four groups as follows:

— Group A contains the most known hydrogen bonds X^…^H–Y (electronic supplementary material, table S7). In our case, X = O or I and Y = C or N.
— Group B contains their N^…^H–Y analogues (electronic supplementary material, table S8) with Y = C or N where the N^…^H interactions could be mediated by π electrons in the sp^2^ hybridized N atoms.
— Group C consists of typical π^…^X interactions (electronic supplementary material, table S9) that are mediated by π electrons at the sp^2^ (or sp)-hybridized C atoms within the C^…^H–C or C^…^O interactions in our case.
— Group D contains the remaining weak interactions, which are presented in electronic supplementary material, table S10. Most of them belong to the least known hydrogen–hydrogen bonding [80] of the C–H^…^H–C type between similar H atoms. We also include here O...O and sp^3^-hybridized C^…^H–C interactions.

**Figure 9.**
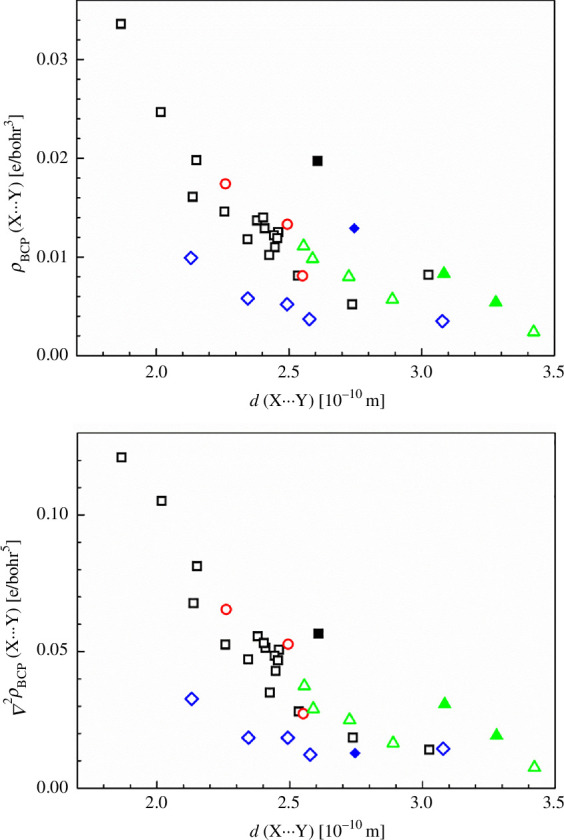
Upper: relation between bond critical point (BCP) electron density, *ρ*_BCP_(X^…^Y), and bond length, *d*(X^…^Y), of the weak interactions X^…^Y in groups A–D of the compounds under study (A—black squares, B—red circles, C—green triangles, D—blue diamonds, I^…^H interaction in BECNUV—filled square, O^…^C interactions in RUCBEZ—filled triangles, O^…^O interaction in OKEWEG—filled diamond). Lower: relation between the Laplacian of the BCP electron density, ∇^2^ *ρ*_BCP_(X^…^Y) and bond length, *d*(X^…^Y), of the weak interactions X^…^Y in the groups A–D of the compounds under study (A—black squares, B—red circles, C—green triangles, D—blue diamonds, I^…^H interaction in BECNUV—filled square, O^…^C interactions in RUCBEZ—filled triangles, O^…^O interaction in OKEWEG—filled diamond).

As expected, the above weak interactions are significantly longer than usual chemical bonds and thus have *ca* 1–2 orders lower BCP electron density. In general, the decrease of the BCP electron density of weak bonds with the increase in their length is nearly linear (see figure 9). The exceptions are hydrogen bonds I1^…^H5 in BECNUV (iodine is much heavier than the O, N and C atoms) and O3^…^H16 in RUCBEZ (this bond path is longer than 3 Å and probably does not have bonding character). The same trend holds for the Laplacians of BCP electron density (see figure 9), again except for the hydrogen bond I1^…^H5 in BECNUV. Independent of the weak type of weak bonds, their ∇^2^ *ρ*_BCP_ is always positive (closed shell bonding [45]). Under *ca* 120^o^, the increase of BCP ellipticity of X^…^H bonds with decreasing X-H-Y angles is observed in hydrogen bonds X^…^H–Y and their N^…^H–Y analogues (A and B groups) only. The instability of some weak bonds (especially of the C group) is reflected in their extra-high BCP ellipticity values. In agreement with the literature [80], hydrogen–hydrogen bonding (C–H^…^H–C type; see electronic supplementary material, table S10) is connected with longer H–C bonds than in classical hydrogen bonding of O^…^H–C type (see electronic supplementary material, table S7 and figure S11). For each type of bonding interaction, the absolute values of X^…^Y bond energies |*E*_b_| decrease with increasing X^…^Y distances. Hydrogen bonds O^…^H and N^…^H (A and B groups) have the highest |*E*_b_| values (electronic supplementary material, tables S7 and S8), while the lowest values correspond to the π^…^X interactions (D group with substantially higher C^…^H distances; see electronic supplementary material, table S10 and figure S11). The chemical bonds H–C are longer, with lower *ρ*_BCP_ and less negative ∇^2^ *ρ*_BCP_ values than the H–N bonds. In both cases, their BCP ellipticity is small. A comparison of the electron density at RCP, *ρ*_RCP_, of five-membered and six-membered rings in the molecules under study, which are *ca* 1 order lower than *ρ*_BCP_ values of the usual chemical bonds, is presented— specifically, *ρ*_BCP_ values of the H–C bonds in electronic supplementary material, tables S7–S11. Moreover, *ρ*_RC_ and *ρ*_RCP_ values of the six-membered rings are about half of those of the five-membered ones. This substantiates why the π^…^X interactions cannot be directed to the centroids Cg of aromatic rings. The Cg–X distance serves as an estimation of the X distance to the single interacting ring atom only.

**Figure 10.**
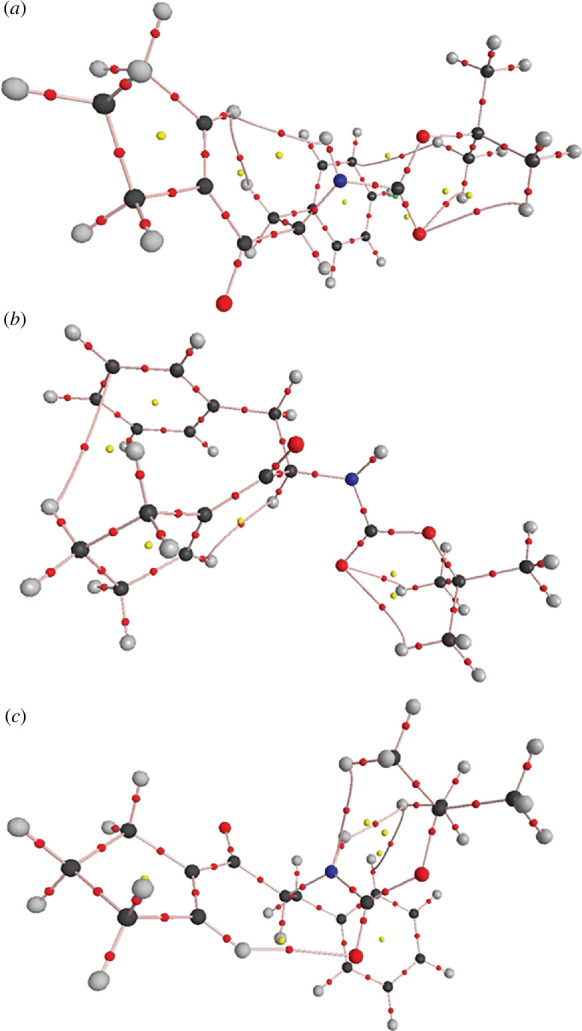
Molecular graph of *(a*) **1**-A1, (*b*) **1**-A2 and (*c*) **1**-A3. For all isomers: C—black, H—grey, O—larger red, N—blue, bond critical point (BCP)—small red, ring critical point (RCP)—yellow.

In the next step, we modified the structure of **1** to break its weak intramolecular interactions O2^…^H4, O2^…^H173/H181 or C12^…^H183 (the corresponding dihedral angles were changed by 180^0^). After optimization, its alternative isomers **1**-A1, **1**-A2 and **1**-A3 (figure 10) were obtained with new frameworks of weak intramolecular interactions (electronic supplementary material, tables S7–S12). The electron density at the RCP, *ρ*_RCP_, is practically the same for all **1** isomers. The total bond energies *E*_b_ of their weak interactions are nearly equal despite changing their types. The energy differences between individual **1** isomers are lower than or comparable to their total |*E*_b_| values. This indicates that the distribution of weak intramolecular interactions within a molecule is more important for the molecular geometry than their bond energies.

### 3.5. Bio-screening

Having studied the (supra)molecular features of the analysed compounds in the solid state, we moved on to look for correlations in a biological setting. It can be mentioned that the World Health Organization (WHO) report [80] addressed the global burden posed by cancer, with more than 18.1 million cases annually. What is more, this is expected to double by 2040 [81]. Breast cancer is the most common malignancy among women and thereby the world’s second leading cause of cancer-related death (*ca* 25% of all reported cancers) [82–85]. Despite the advances in developing novel medications, tumour resistance to the treatment is still reported. Therefore, further research is needed to find more effective and safe lead molecules. It should be highlighted that peptide compounds have great promise in anticancer therapy. What is more, according to the literature, cyclopentene plays a role in the inhibition of breast cancer [86–88].

#### 3.5.1. Bio-pharmacokinetic properties

The pharmacokinetic profile, including molecular weight, polar surface area, number of donors/acceptors, molecular refractivity and lipophilicity, i.e. partition coefficients such as log*P*_o_/*w* of *n*-octanol, and WLOGP, MLOGP and XLOGP3 [89] of the analysed compounds, are presented in electronic supplementary material, tables S13 and S14. Overall, the compounds reveal the potential for drug-likeness, safety and cell permeability. Among all the analysed compounds, **1**, BECNUV and TOBSUA satisfy criteria for lipophilicity, flexibility, polarity, size, unsaturation and insolubility; see Bioavailability Radar charts (figure 6; electronic supplementary material, figure S12). They can pass the blood–brain barrier. Based on the *in silico* predictions and generated BOILED-Egg (brain or intestinal estimated) model, it can be concluded that the new chemical compound **1** has good drug-likeness as regards intestinal absorption (topological polar surface area) (figure 6; electronic supplementary material, figure S13).

In addition, possible molecular intracellular targets determined using SwissTargetPrediction [90] are depicted in electronic supplementary material, figure S14. The new compound, based on its interaction with selected main receptors, is a potential inhibitor of protease (index 0.73), an enzyme (0.38), a ‘nuclear receptor ligand’ (0.22) and a G protein-coupled receptor (GPCR; 0.30).

Moreover, the new compound was tested *in silico* in the context of cytotoxicity towards cancer cells, based on the PASS procedure, on the CLC-Pred server [91]. It is interesting to observe relatively good potential against cancer cells of the breast, but lower potential against cancer cells of the pancreas and haematopoietic system has been revealed (electronic supplementary material, table S15).

#### 3.5.2. Molecular docking protocol

Protein kinases are crucial enzymes that regulate nearly all cellular processes [92]. Unfortunately, their dysregulation is implicated in various diseases, including cancer, autoimmune disorders and degenerative dysfunctions. Among 500 protein kinases encoded by the human genome [93], serine/threonine kinases such as casein kinase 2 (CK2) [94] and proviral integration of Moloney virus-1 (PIM-1) [95] kinase are major drug targets. This trend in modern medicinal chemistry can be attributed to the fact that the overexpression of CK2 and PIM-1 occurs in many types of cancers, including breast, leukaemia and prostate cancer [96–100]. In the last few years, significant progress has also been made in the development of dual CK2/PIM-1 inhibitors, resulting in the discovery of novel small-molecule active agents exhibiting improved cytotoxic potency and specificity, especially towards breast cancer cell lines *in vivo* [53,101–103].

**Figure 11.**
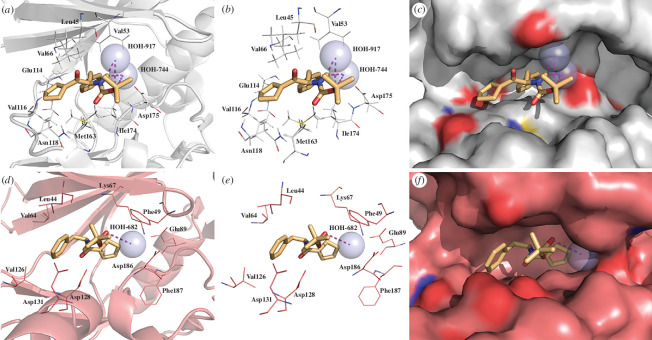
Representative three-dimensional binding modes of **1** with human protein kinases CK2-α (PDB code: 4KWP; (*a*–*c*)) and PIM-1 (PDB code: 4DTK; (*d*–*f*)), including close contacts with critical amino acid residues in the ATP-binding sites of both receptors.

To better understand the potential biological activity of the developed agent **1** at the molecular level, three-dimensional models of both proteins mentioned above in complex with this ligand were generated. In this regard, the substrate binding modes were predicted by flexible docking calculations performed with the AutoDock Vina program, employing the crystal structures of human protein kinases CK2-α (PDB code: 4KWP) [50] and PIM-1 (PDB code: 4DTK) [51], downloaded from the Protein Data Bank (PDB) (figure 11). For validation of the docking protocol and the subsequent in-depth analysis of the docking results in terms of the binding energies and, thus, the mutual ligand–receptor affinities, a clinically used CK2 inhibitor, namely Silmitasertib (C**1**-4945) [104], was docked as a control ligand (for more details, see the electronic supplementary material). The maximum docking score of **1** in CK2**-**α was −7.3 kcal mol^−1^, while in PIM-1, the absolute free binding energies (Δ*G*_calc_) reached up to −8.3 kcal mol^−1^. The obtained results showed that **1** exhibits relatively high potency to form stable complexes with both kinases even when compared with the employed CX-4945, which attained Δ*G*_calc_ values of −8.9 kcal mol^−1^ for CK2**-**α and −9.4 kcal mol^−1^ for PIM-1, respectively.

Figure 11 shows the docking results, depicting the intermolecular interactions between **1** and the critical amino acids of CK2**-**α and PIM-1 in their catalytic cavities.

Inspecting the most energetically favourable binding mode of **1** in CK2**-**α (electronic supplementary material, figure S16 and table S16), we found that this compound forms strong 2.2−2.3 Å hydrogen bonds with two conserved crystal water molecules, namely HOH-744 and HOH-917. At the same time, its benzene ring is located deep inside the ATP-binding pocket, thus exhibiting hydrophobic π-alkyl interactions with Ile174 and Met163 and π–σ interactions with Val66, respectively. Furthermore, the cyclopentene moiety of **1** establishes alkyl CH–CH vdW interactions with hydrophobic amino acid residues, such as Val53, Lys68 and Leu45.

In turn, molecular docking showed that the critical interactions between **1** and amino acids at the active site of PIM-1 included π−σ dispersion forces involving the benzene ring and Leu44, π−alkyl interactions in the case of the electron-rich aromatic ring of **1** and aliphatic residues of Leu174 and Val126. Other beneficial ligand–receptor interactions potentially responsible for stabilizing the **1**−PIM-1 complex include the alkyl-type interactions observed between the carbon atoms of the ligand cyclopentene ring and the aliphatic amino acids of the receptor (i.e. Leu120, Ile104, Ile185 and catalytic Lys67). Moreover, the oxygen atom of the carbamate moiety of **1** formed a 3.4 Å hydrogen bond with crystal water (HOH-682).

In conclusion, molecular docking revealed that in both studied complexes of **1**, with CK2-α or PIM-1, a ligand molecule **1** is firmly positioned in the ATP-binding pocket, and dispersion forces play a decisive role in stabilizing those complexes. Moreover, owing to a relatively narrow tunnel-like architecture of the substrate binding pockets of both studied kinases, the ligand molecule is forced to adapt a linear (open) conformation in complex with those receptors. Hence, weak intramolecular interactions between peripheral rings of **1** and the oxygen atom of its carbamate moiety were here surpassed via intermolecular contacts to avoid the repulsive steric clashes between the sterically hindered bulky *tert*-butyl substituent and amino acid residues of the ATP-binding pockets of both kinases.

Notably, compound **1** has shown promising results *in silico* as a potential inhibitor of CK2 and PIM-1. Thus, it can be considered a perfect starting point for subsequent drug discovery for kinase targets and breast cancer management.

## 4. Conclusion and perspective

In summary, the synthesis and the crystal structure of (*R*)-(2-*tert*-butoxycarbonyl)amino-1-oxo-3-phenyl)propyl)-1-cyclopentene (**1**) as well as the detailed supramolecular characterization of **1** and similar compounds derived from the CSD are reported. The novel compound crystallizes in an orthorhombic crystal system with a *P*2_1_2_1_2_1_ space group with two independent molecules with a similar conformation in the asymmetric unit. H-bonding self-assembled supramolecular units generate a zig-zag chain-like structure forming intermolecular interactions resulting in the formation of diverse intraolecular (S-type) and intermolecular (D-, C-type) H-bonding supramolecular patterns. A library of supramolecular synthons is provided. X-ray crystallography revealed that cyclopentene shields peptide bonds via intramolecular C–H^…^O interaction, leading to a pseudo-cyclic motif denoted *S*(8) according to graph-set theory. In addition, the conformation is stabilized through intramolecular C–O^…^π contact in which the phenyl group of phenylalanine is involved. In consequence, a unique cyclopentene-based pseudo-bicyclic system shielding an amide bond is observed for the first time, which increases the value of the novel compound. Moreover, it sheds new light on cyclopentene as well as other similar bioisosteres shielding an amide bond in designing ‘molecular chameleons’; the amide bond could be more resistant to cellular metabolic proteolysis. A multi-method approach, including DFT, QTAIM, FIM and complex HS analysis, together with molecular EP, intermolecular interaction energies and EF as well as enrichment ratio, provided more profound insights into the nature of weak interactions such as O(N,C,S,I)^…^H/H^…^O(N,C,S,I), C^…^C, O^…^O, O(N,I)^…^C/C^…^O(N,I), confirming the significance of the cooperative effects of such interactions in crystal packing of cyclopentene-containing peptide derivatives. According to the HS analysis, in the crystal packing of novel compound **1**, the main contributors are H^…^H contacts (74%), H^…^O/O^…^H (13.5%) and C^…^H/H^…^C (11.5%). In addition, N^…^H/H^…^N, C^…^C and C^…^O/O^…^C are noticeable. Nevertheless, according to the enrichment ratio calculations, only H^…^O and C^…^H are preferable. EF calculations revealed larger values of dispersion energy, indicating the dominance of weak noncovalent interactions over hydrogen bonding interactions in terms of electrostatic forces in crystal packing of either novel compound **1** or similar discussed structures retrieved from the CSD. The crystallographic studies established the cyclopentene as an important supramolecular tecton and a widespread contributor in either intra- or intermolecular H-bonding and π-based intercontacts. It plays the role of either acceptor or donor, forming synthons at diverse levels of supramolecular architecture. QTAIM topological analysis of electron density can rationalize all types of weak bonds in molecules and crystals. Their characteristics exhibit many similarities for all types of weak bonds. We have shown that weak intramolecular interactions significantly contribute to the relative stability of individual conformers. Their importance is based more on their suitable distribution within a molecule than on their energy contributions. In addition, extended HS analysis, including EF, as well as molecular docking studies, revealed the significance of dispersion forces in the architecture of ligand structure and ligand–protein bio-complex, respectively. Notably, cyclopentene forms beneficial ligand–receptor interactions, thus strengthening binding affinity in the studied complexes. The docking protocol allowed us to establish the potential of **1** against breast cancer. *In silico* bio-physicochemical, ADME-T and drug-likeness properties of **1** were found to be satisfactory, fulfilling ‘Lipinski’s rule of 5’.

Taken together, examination of **1** in various environments reveals flexible shielding of a peptide bond via the ability to form dynamic intramolecular interactions with cyclopentene. The findings presenting the multifaceted nature of cyclopentene, a bioisostere of proline, portray structural guidance in pharmacophore design in the context of modulation of conformation via noncovalent intramolecular interactions, and open new perspectives in the search for novel smart modified peptide-based molecules more effective in the treatment of cancers.

## Data Availability

Data are provided in the online supplementary material [105].
